# Auxiliary Companies of the Horticultural Sector as a Competitiveness Element: The Case of Almeria (Spain)

**DOI:** 10.3390/ijerph16142575

**Published:** 2019-07-18

**Authors:** Jaime de Pablo Valenciano, Juan Uribe-Toril, Juan Milán-García, José Luis Ruiz-Real, José Antonio Torres Arriaza

**Affiliations:** 1 Faculty of Economics and Business, University of Almería, 04120 Almería, Spain; 2 Faculty of Computer Science, University of Almería, 04120 Almería, Spain

**Keywords:** Almeria, auxiliary company, greenhouses, competitiveness, sustainability, extrapolation

## Abstract

The horticultural model of Almeria (Spain) based on the operation of greenhouses is an international reference and has been considered as an economic miracle. Alongside this agricultural development has been the deployment of the diverse productive activity of auxiliary companies. The objective of this article is to understand how these companies operate and analyze their factors of competitiveness, competing needs, and future competitive improvements, taking as reference four of the most important subsectors (machinery, greenhouse infrastructure, plastics, and seeds). The Delphi method was used and through a panel of experts the conditioning factors of each of the variables to be analyzed (factors, needs, and competitiveness improvements) was chosen. Of the 120 companies that were sent questionnaires, 72 participated. The sectors that make up the auxiliary companies are heterogeneous and therefore the results obtained have differed among them. The synergies between the greenhouse crops and the auxiliary companies are an example of diversification of productive activity that can be extrapolated to other production areas worldwide. The future of the auxiliary companies is linked to that of the intensive agriculture and the key variables must be underscored by competitiveness and sustainability.

## 1. Introduction

**Figure 1:**
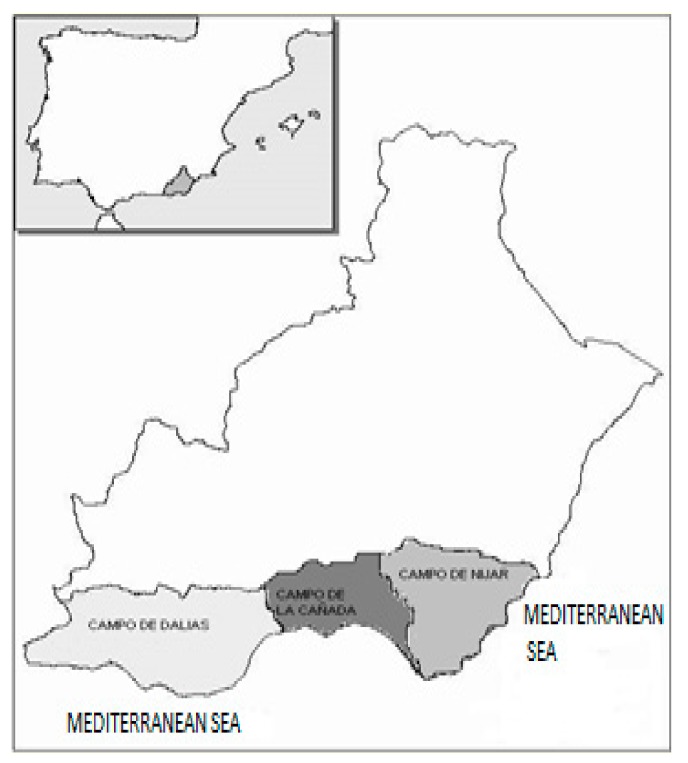
Location of Almeria and its productive zones of cultivation under plastic.

The province of Almeria is situated in Southeast Spain (Figure 1). Its horticultural sector, based on the operation of greenhouses, is an international reference. It is the leading province in the production and export of many vegetables (tomato, pepper, eggplant, zucchini, watermelon, and melon) of the European Union.

The principal production zones are located in the central region of the province, in the Campo de Dalias, and to a lesser extent, in la Cañada and Campo de Níjar.

**Figure 2:**
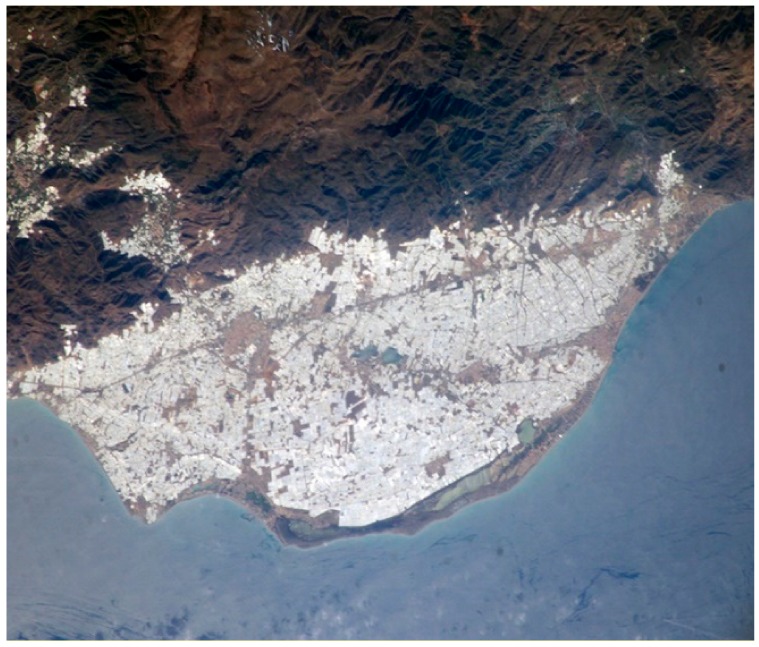
Sea of plastic.

The horticultural sector of Almeria has been considered as an agricultural miracle [1,2,3,4,5]. Indeed, from the air, it is more than 23,000 greenhouses that resemble a sea of plastic as can be seen in Figure 2 [6,7,8,9].

This agricultural model is the result of the combination of a series of factors including optimal climate, entrepreneurship of farmers, technologies, and financing through credit unions. It began in 1941, with the declaration of the Campo de Dalías as an area of national interest. The general plans of colonization and transformation appeared from 1953 onward (1953, 1958, 1964, 1969, 1970, 1971, 1973, 1977, and 1982) in order to enable new irrigation regimes that can benefit from existing water in the subsoil [10].

This model has been extrapolated to other production zones, such as in Latin America, as reflected in the document, *The Protected Crop in Mediterranean Climate*, carried out by the Food and Agriculture Organization (FAO) [11]. The model has been implemented in Mexico, specifically, in Baja California Sur, Guanajuato, Jalisco, State of Mexico, Sonora Yucatan [12], and Sinaloa [13]; in Colombia in the mid-Sinú Valley [14] and in Boyacá, Cundinamarca, Santander and Valle (Colombia); and in Chile in the Arica region.

The horticultural sector has gone from being run by small family enterprises to industrial-size operations, and an important auxiliary industry has developed alongside it. The pioneering work of auxiliary companies in Almería was carried out by the Andalusian Development Institute [15], which in 1989 drew up the “Global Action Plan for the Auxiliary Industry of the Almerian Poniente.” Later, in 2007, the Institute of Studies of Cajamar [16] published the report “Plan for the promotion of the productive system linked to agriculture in Almeria.” In addition, the Foundation for Agrarian Research of the Province of Almeria [17] and the Tecnova technology center [18] have also drafted two strategic plans (2006 and 2009).

The need to implement a rural policy for the diversification of productive activity, such as the implementation of an auxiliary industry for fruit and vegetable production, has, as a consequence, resulted in the endogenous development of the Campo de Dalías region. The result is that a significant auxiliary industry is currently being consolidated with an important part of its products being dedicated to the export sector.

The concept of an auxiliary agricultural company brings together a large number of activities of various kinds that have a common purpose in serving as suppliers of inputs to intensive agriculture. Table 1 shows which sectors makeup this auxiliary sector.

**Figure 3:**
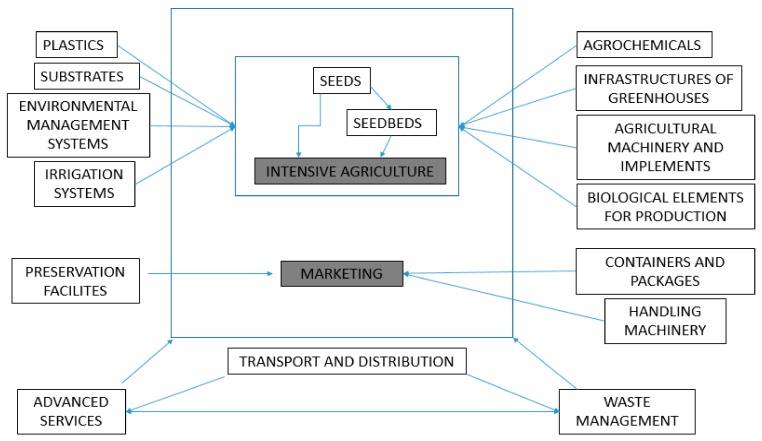
Relationship of auxiliary companies with intensive farming.

The inter-relationships between intensive farming and the various sectors that makeup the auxiliary industries are presented in Figure 3.

This integration process of auxiliary companies in agriculture can be considered to be one more step toward the consolidation of the cluster of horticultural companies in a global market where competitiveness and innovation are key factors for its proper functioning. Authors have given several definitions to the concept of business cluster. The one that is used in this article is the definition given by Porter [19]: “Clusters are geographic concentrations of interconnected companies, specialized suppliers, service providers, firms in related industries, and associated institutions (for example, universities, standards agencies, and trade associations) in particular fields that compete but also cooperate.” More recent studies make a classification of kinds of business cluster such as the work of Sforzi [20].

The purpose of this article is to analyze the competitive factors, competing needs, and future improvements of the most representative companies in the auxiliary industry sector in the intensely farmed area of Almeria (infrastructure of greenhouses, machinery, plastics, and seedbed cultivators). The article can serve as an example to other production areas, where the auxiliary industry may still be in its infancy.

## 2. Material and Methods

The Delphi method was developed by Olaf Helmer Norman Dalkey and Nicholas Resche in 1959 [21,22] and has been used with several modifications and reformulations since then [23].

According to Linstone and Turoff [24], the Delphi method aims to structure the group communication process in order to facilitate a group of individuals to deal with complex problems as a whole.

This method is used to analyze the opinions of a group of experts, who remain isolated in order to minimize the effect of social pressure and other aspects of the behavior of small groups. The experts can be internal or external specialists. When deploying the Delphi method it is customary to follow a certain sequence, but there is no rigid structure. It is based on the Brainstorming technique. That is, a compendium of ideas and strategies contemplated by different people who face a topic.

In order to avoid the problems that arise from face-to-face encounters, the Delphi method resorts to the anonymity of the different members of the group and that of individual responses. This is guaranteed by the evaluation of questionnaires, since the set of responses of the participants (including minorities) is considered as the results of the exercise.

The evaluation of the questionnaires is done in such a way that the results can be incorporated as additional information to the questions of successive questionnaires (feedback). This allows the participants of the Delphi exercise to review their proposals in light of new information that is delivered to them.

**Figure 4:**
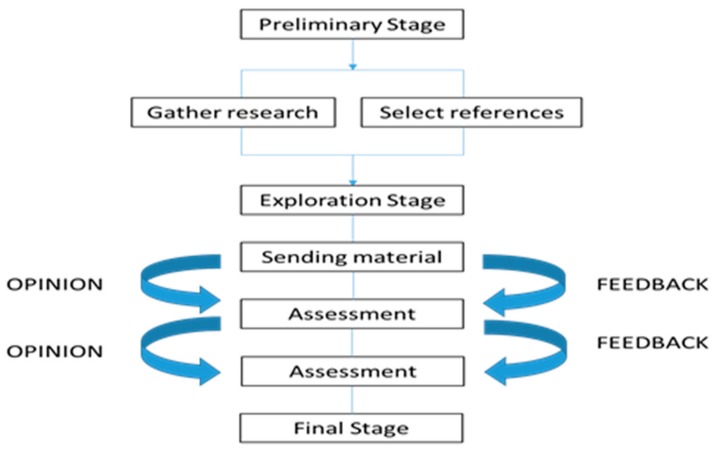
Description of Delphi phases.

It can be characterized, as seen in Figure 4, by three phases: preliminary, exploratory, and final.

In this research, related to the competitiveness of the different sectors of auxiliary companies, the following steps were completed. In the preliminary phase of Delphi, the context, objectives, and the basic elements of the evaluation are defined; the reference group to be consulted is selected, and the research works with references to the available methodologies to analyze the reference group are consulted. In this instance, of the 120 companies that were sent questionnaires, 72 participated.

In the exploratory phase, the collected studies are sent to the group so that the answers on the evaluation of effectiveness and efficiency of each indicator and following an initial consultation are agreed upon in the next round. For the purposes of this research, the following factors were analyzed:

(a) The competitiveness factors that reflect the critical elements for the sector;
(b) Competing needs, that is, those actions that are considered urgent and taken as a reference;
(c) The improvement of competitiveness, being actions aimed at increasing the competitiveness of the local productive model.

The conditions that exert the greatest influence on the factors and needs as well as those that improve competitiveness, as indicated by the expert group are indicated in Table 2.

**Figure A1:**
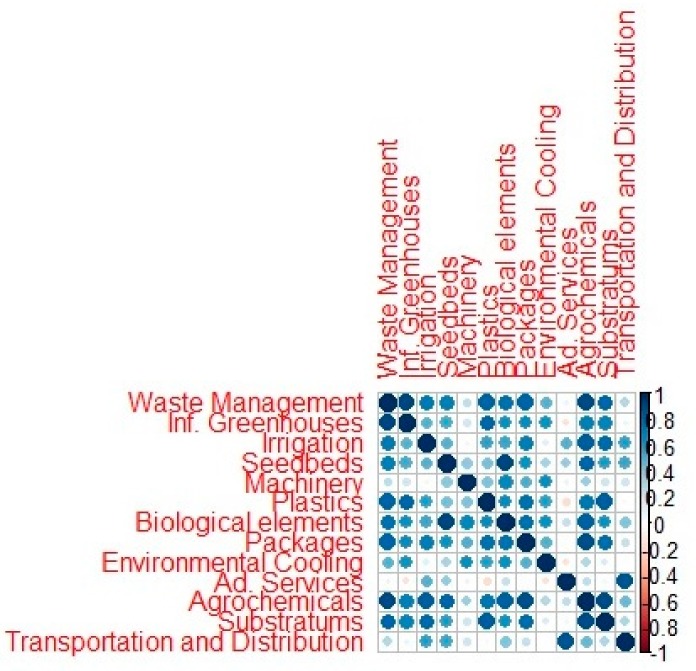
Correlation analysis of competitiveness factors.

**Figure A2:**
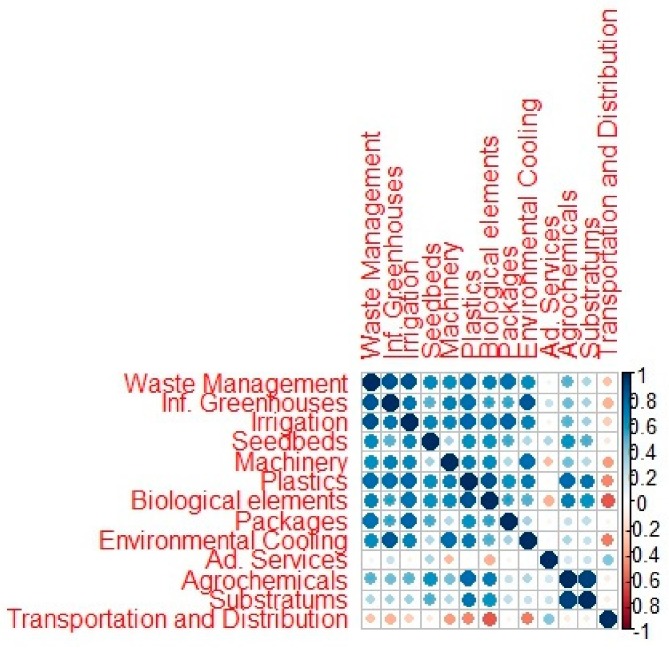
Correlation analysis of competing needs.

**Figure A3:**
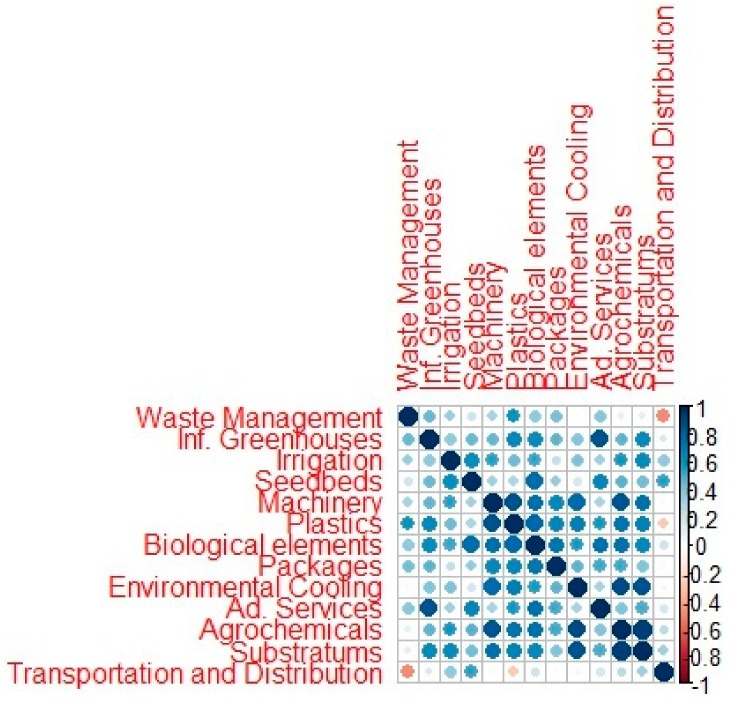
Correlation analysis of improvement of local competitiveness.

In the final phase, a comparative table reflecting a balance of opinions on the perceived evaluation on each indicator was compiled, where 10 is the most relevant and 0 the least (see Appendix A: Table A1, Table A2, Table A3 and Table A4 and Figure A1, Figure A2 and Figure A3).

Among the advantages of the Delphi method, the following are worth mentioning: (a) It is a fast and efficient system for obtaining objective information from a group of experts, specialists, or referents; (b) it is a simple method that facilitates the agreement between several opinions; (c) it can be considered as another instrument in the acquisition of information on the perception and analysis of a particular topic; (d) it is a structured method that facilitates the construction of an analysis, incorporating different variables to analyze in each consultation phase.

## 3. Results

### 3.1. Infraestructure of Greenhouses

This sector is made up of all those companies that provide plans, designs, and construction of metal and plastic structures for the construction of greenhouses and industrial buildings. This is in addition to the ground preparation for laying the foundations destined for diverse agricultural constructions; the provision of services, including the installation of electromechanical equipment for the operation of the different components of the greenhouse; irrigation and fertilization systems, which monitor the humidity and temperature within these structures.

**Figure 5:**
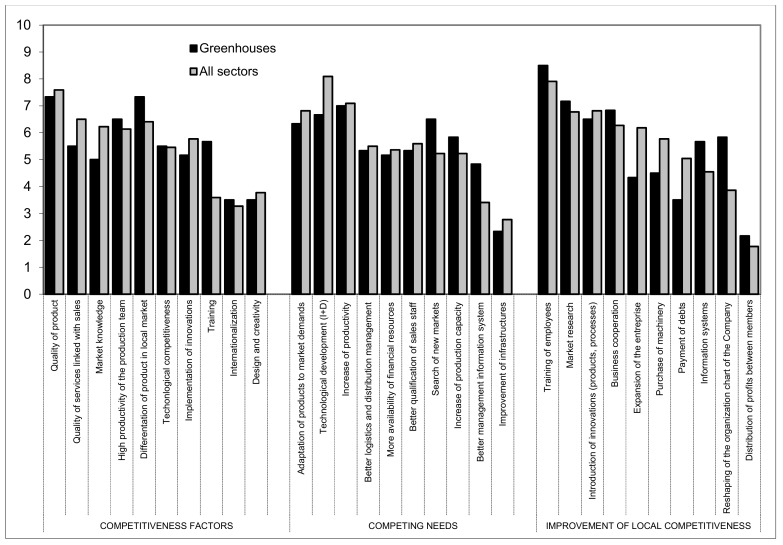
Issues related to competitiveness of the infrastructures of greenhouses with regards to other subsectors.

A total of 15 companies of this sector participated in the analysis (Figure 5).

According to the research, the three most important competitiveness factors are: Quality of product (7.33), product differentiation in the local market (7.33), and a high level of productivity by the production team (6.5). The quality of the products produced leads to differentiation in the local market, while a high level of productivity means that the producer can charge a lower price per meter square of the greenhouse.

With regards to the competing needs, the results are focused on an increase in productivity (7), technological development (6.67), the search for new markets (6.5), and product adaptation to current market needs (6.33). Some of the companies surveyed export to external markets such Morocco and Mexico.

In relation to prioritizing the investment of resources that will improve competitiveness, the results highlight training (8.5), market research (7.17), business cooperation (6.83), and introduction of innovations; products, processes, etc. (6.5).

A focus on the future needs of the sector, this strategy can involve several stages; a deepening in the internationalization of the sector seeking greater profitability. In this case, companies must adapt their product/service to new weather conditions and new production needs. This potential stage will require companies to have a high levels of Research and Development (R&D) costs for the innovative and new geometric designs of greenhouses that guarantee and optimize the production, the rethinking of the procedure of irrigation, fertilization, and climate control in the new greenhouses and the development of communication strategies with the client based on Information and Communications Technologies (ICTs) that optimize technical and after-sales service. Consumers grant greater value to those companies with which they can interact directly and have their problems solved in a quick, efficient, and transparent manner, generating long-term relationships of trust [25].

### 3.2. Agricultural Machinery and Tools

This sector is comprised of the companies that supply agricultural machinery and tools to be used in greenhouses for tasks such as planting, harvesting, pruning, autonomous irrigation, and other tasks, as well as those that offer industrial products for packaging, quality control, and handling in the storage of products.

**Figure 6:**
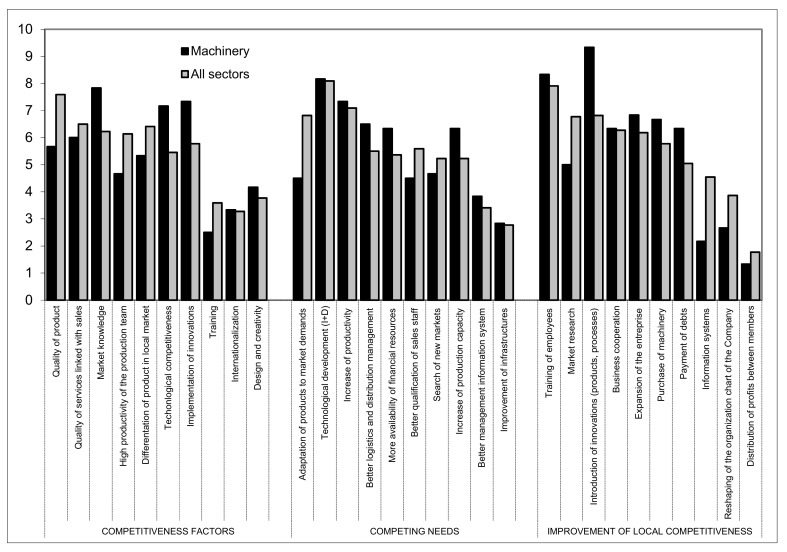
Issues referring to competitiveness of agricultural machinery and tools in relation with other subsectors. Source—Own compilation based on data from Delphi analysis and surveys.

A total of 26 companies of this sector participated in the analysis (Figure 6).

The key factors of competitiveness in this sector are market knowledge (7.83), implementation of innovations (7.33), and technological competitiveness (7.17). By market knowledge, the experts refer to a constant presence in the client’s premises, paying attention to their needs which in turn means that the client can help with the implementation of innovations. Care must be taken to not over-emphasize technological innovation as it can sometimes detract from commercial development. As a result, many viable technologies are not commercialized due to lack of marketing or adequate business skills [26].

In the case of machinery, this is a very restricted to the local market despite the possibilities of exporting to other countries. Some companies in Almeria are already installed in Mexico.

Technological development (8.17), increase in productivity (7.33), better management of logistics and distribution (6.5) and greater availability of financial resources (6.33) are the most valued competing needs.

In terms of investing resources to improve competitiveness, the introduction of innovations (9.33), along with training (8.33) are considered the basis of the future. Less value is given to the expansion of the company (6.83), acquisition of machinery (6.67), payment of debts (6.33), and business cooperation (6.33). The introduction of innovations permits customers to lower their production costs and thus convince them to buy their machinery.

The most important innovations that this subsector can generate are in the reduction of operating costs (mainly labor) in the operation of greenhouses.

This group of auxiliary companies has an inherent need for innovation that can offer them a competitive advantage over its rivals. They are, however, faced with several challenges.

One of these challenges is the manufacture of machinery for quality control, both perceived and using non-destructive methods, to determine the internal quality of the product. This technology is already being offered as a service in specific applications and it is expected that its use will be extended to all product ranges. This will signify benefits in terms of standardization of quality, improvement in the perception of quality on the part of the customer, and reduction in operation costs [26,27,28].

The development of automation and robotic technology is necessary to intensify greenhouse production [29]. This technology is already being offered as a service in specific applications, and it predicted that its usage will be extended to all product ranges due to the benefits in quality standardization, the improved perception of quality on the part of the customer, and the reduction of operative costs [30].

The ability to sell in the international markets requires new ICT services for tele-maintenance and tele-operation.

### 3.3. Plastics

The plastics chemical industry in Almeria can boast several benchmark companies in the production of plastic roofs for greenhouses. The services offered by the plastics industry range from the roof, through to the padding and floor disinfection matting. These industries bank on the incorporation of new functionalities, performance standards, and utilization in agriculture.

**Figure 7:**
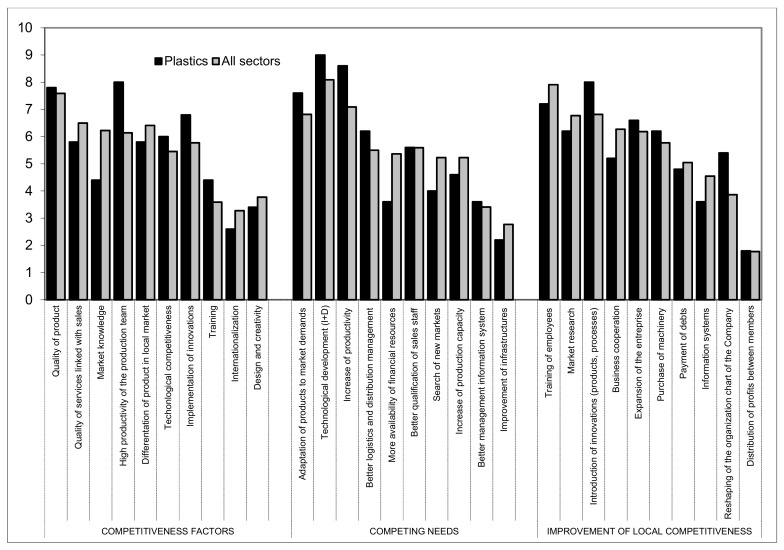
Issues related to competitiveness of the plastics sector in relation with other subsectors. Source—Own compilation based on data from Delphi analysis and surveys.

Nine auxiliary companies in this sector participated in the analysis (Figure 7).

The most valued competitiveness factors of this sector in order of importance are: High levels of productivity of the production team (8), product quality (7.8), implementation of innovations (6.8), and technological competitiveness (6). This may be due to international financial crisis that we are currently experiencing which, in turn, has generated many problems of non-payment together with a generalized increase in costs.

With regards to competing needs identified within the plastics subsector of auxiliary companies, the following can be highlighted: technological development (9), increase in productivity (8.6), adaptation of the product to market requirements (7.6), and better logistics management and distribution (6.2).

Technological development and the increase of added value are the biggest challenges of this sector. The only way to face the exporting and marketing intensity of countries such as China or India is by specializing in the production of products with higher added value. Another problem that this sector must face is the small scale of the companies, which makes the application of innovations more difficult. A consolidation of companies may become essential in order to access greater economies of scale and strengthen technological development [31].

In the interest of investing resources which would optimize competitiveness, the introduction of innovations (8) and training of workers (7.2) are highly valued, followed by company expansion (6.6), acquisition of machinery (6.2), and market research (6.2).

Extending product functionality is a direct consequence of the cost of innovation, which is producing more resistant plastics which are partially transparent to certain radiations, with certain wavelengths (to filter, for example, ultraviolet rays and let pass infrared rays), with chemical properties that favor certain degrees of humidity as well as new products to be used in collateral operations inside the greenhouse.

It is expected that in the short and medium term, there will be new plastics manufactured that will use radiant energy, be more resistant, and with bactericidal and fungicidal properties.

### 3.4. Seedbed Cultivators

The seedbed cultivator sector is dedicated to the development of seeds via the study, selection, crossing, and improvement of agricultural products using hybrid varieties that present characteristics of form, variety, and external texture, accepted by the final client and that present in turn elements of intrinsic quality (sugar, acidity, consistency of the pulp) in accordance with the quality standards accepted by the markets. These types of hybrid are, in some cases, adapted to adverse weather and certain resistance to some pathogens that can degrade or destroy the fruit. Thus, as can be seen, innovation in this field is the raison d’etre of these companies. For these companies, hybridization techniques and the expertise in these are essential to their operation. In this sector, the debate about the use of genetic manipulation techniques to obtain new product varietals remains open. These types of processes, however, may clash with the laws within the consumer countries, thus restricting the use of genetic manipulation techniques.

Innovation should not be limited to mere technical terminology, but should be understood as an economic, social, organizational, and strategic phenomenon that changes the established procedures and ways of doing things [32,33].

The process that leads to innovation requires specialized knowledge, organization, and financial support, which is why most of the R&D expenditure is found in large companies.

The seedbed cultivators or nurseries offer the rapid growth of seeds as a service in order to attain useful seedlings for the subsequent planting in greenhouses. This process is more profitable than planting the seeds directly in the greenhouse. The seedbed cultivators specialize in achieving the optimum conditions in terms of humidity, temperature, and hygiene necessary to achieve a quality seedling in relatively short times. The challenges in innovation facing these types of industries have to do with:

(a) The eradication of plagues by viruses in the seedlings that once planted in the greenhouses, generate epidemics in the crops that significantly damage the production. The R&D studies on the origin, spread, and fight against pathogens in seedlings will have an immediate impact on the profitability of the seedbed cultivators.
(b) The control of the work being carried out on the different seedlings and the exhaustive monitoring of the operations of these through advanced processes of traceability. The intensive use of ICT technologies and smart labeling systems will support this process.
(c) Innovation in techniques and the study of plant biology to improve germination times and achieve a greater volume of good quality seedlings will always be a fundamental pillar of this activity.

**Figure 8:**
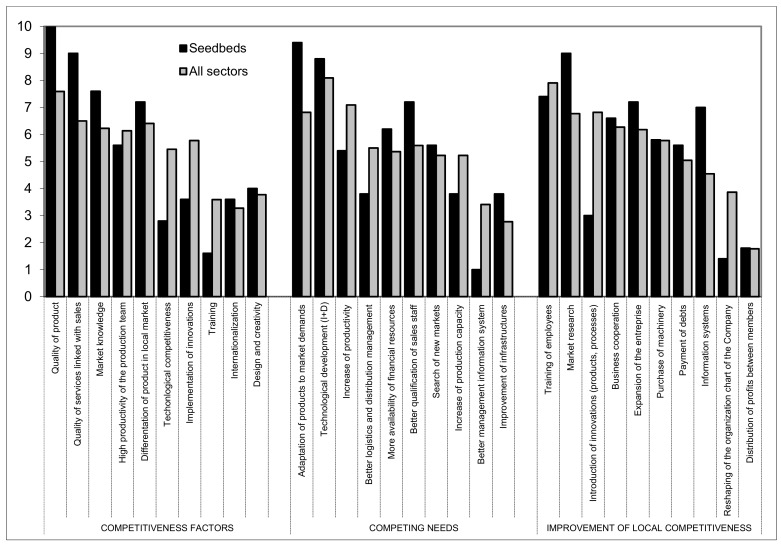
Issues referring to competitiveness of seedbed cultivators in relation with other subsectors. Source—Own compilation based on data from Delphi analysis and surveys.

A total of 22 companies in this subsector of auxiliary companies took part in this analysis (Figure 8).

The most representative competitiveness factors for these auxiliary companies are: Product quality (10), service quality linked to sales (9), market knowledge (7.6), and product differentiation in the local market (7.2). If the grower needs seeds, then the price is a secondary factor. Though it also the case that there is a very high level of quality customer service related to the sales function.

With regards to competing needs, the most important factors according to the expert group are: Product adaptation to market demand (9.4); technological development (8.8); highly qualified sales personnel (7.2); and greater availability of financial resources (6.2). To this end, multinational seed companies have based their research centers in Almeria.

In terms of the investment of resources to improve competitiveness, the most valued are market research (9), worker training (7.4), company expansion (7.2), information systems (7), and business cooperation (6.6).

Companies of the sector are increasingly aware of the need to adapt their products to market demands. They are better informed owing to the Internet, both in the development of varietals and consumer trends, where consumers are increasingly interested in the origin of the food they purchase. However, representatives of seed cultivators express the growing difficulty of working with decision makers at producer level. Thus, the relation between the geneticist and the producer is becoming increasingly relevant.

Also of note is the low appreciation for issues such as training and the need for replacing qualified professionals and training new staff.

Investment in market and product niche research with higher added value is the basis of growth and consolidation of the seedbed cultivator sector.

Furthermore, Mexico has become a reference in terms of record of fruit and vegetables varietals as a result of the collaboration between administration and producers. Advances achieved allow “safe-manning” to R&D investment, which should improve the international reputation of its agro-alimentary sector [34].

## 4. Discussion

The horticultural model of Almeria is an international reference and focuses on the production of fruits and vegetables in greenhouses [35] and with sustainability being the key variable [36].

It is fundamentally important to diversify the production policies and take advantage of the synergies, as in the case of auxiliary companies that include a heterogeneous number of high technology industries and services [37].

It can be considered that the model of intensive crops and that of the auxiliary companies constitute a cluster [38]. If we apply the definition of Porter [37] there is geographical concentration (Campo de Dalías, Campo de Níjar and La Cañada), specialized providers (auxiliary companies), associated institutions (Universidad de Almería), and financial entities (Cajamar), all of which are interconnected and cooperate with each other.

In order to carry out this study, the Delphi method was used, a valid methodology used in social sciences, as can be seen in numerous studies [39,40,41,42].

Competitiveness is fundamental and therefore it is essential to know which factors promote it, what are the potential competing needs, as well as identify potential future improvements [43,44,45,46].

In the analyzed sectors, greenhouse infrastructure, machinery, and plastics have achieved their own production facilities and even export their products. The same does not apply to the seed suppliers and seedbed cultivators, which are controlled by multinational companies headquartered in the Netherlands and Israel and have their research centers located in Almeria. They play a supporting role as agents for local development because of the lack of local seed research centers [47]. This is due to the lack of capital investment and time spent in R&D processes [48].

It is important to mention that it is possible to extrapolate the synergies between the vegetable crops and auxiliary companies to other production areas, although achieving the combination of these aspects is complicated. However, the demonstrated effect is enriching for the research of other experiences [49].

The productive model of horticultural under plastic as seen in Almeria has been implemented in several Latin American countries such as Mexico [11,12]. This country, in turn, has been a pioneer, and its agricultural production has grown exponentially in the past decade [50]. This notwithstanding, there are significant differences in terms of ownership: Almería is made up of small landowners (an average of two hectares), while in Mexico the production is organized by large landowners.

Mexico faces different obstacles such as the increase in capital cost, the increase in energy costs, inexperience in the management of agricultural companies, the lack of infrastructure and supplies, and the inconsistency in the quality of the product. These obstacles undermine the competitiveness of its auxiliary industry [51].

In an increasingly globalized world, local and regional governments in Latin America must take on new challenges among them those of creating or improving competitive capacities and transforming local productive systems [52]. It must also be borne in mind that it is the companies that are competing, and not the nations [37]. The competing needs of countries, regions, and agents do not necessarily derive solely from the productive resources, but rather from intangible factors such as knowledge, which are the result of developing endogenous competences [53].

## 5. Conclusions

The synergies between the model of intensive agriculture in Almeria and the auxiliary companies that support it is a reality that has brought with it the consolidation of a policy of productive diversification.

These companies are heterogeneous in terms of the use of human resources, capital endowment, and technological level.

However, there is a difference between those auxiliary companies that are focused increasingly on exportation (infrastructures of greenhouses, plastics, and machinery) and those that have a more local focus (seedbed cultivators).

The deployment of auxiliary companies involves the supply of inputs to agricultural operations and a considerable reduction in costs, which in turn improves competitiveness.

In the case of the sectors analyzed: greenhouse infrastructures, plastics, machinery, and seedbed cultivators, the results in relation to competitive factors, competing needs, and future improvements, have been different and reaffirm the heterogeneity of the auxiliary companies.

There are various opportunities related to innovation dependent on the evolution of intensive agriculture and the internationalization capacity of companies. Even so, it is possible to anticipate that the evolution of products and services for intensive agriculture will be accelerated by the appearance of new needs derived from new ways of presenting the products, the need to increase the perceived quality of the products through control techniques and automated quality inspection, the design of new greenhouse structures, together with machinery and procedures that optimize collection tasks and minimize the need for unskilled labor.

The development of auxiliary companies is achievable in other emerging areas of greenhouse production, such as Latin American countries.

The future of the auxiliary companies is linked to that of the intensive crops and the key variables must be marked by competitiveness and sustainability but always bearing in mind that it is a heterogeneous sector and therefore it is necessary to analyze independently the different sub-sectors. The analysis of factors, needs, and improvements in competitiveness must improve productivity with innovative actions aimed not only at the internal market but also at external markets.

## Figures and Tables

**Table 1:** Sectors of auxiliary companies.

Sector	Products
Agrochemicals	Fertilizers, phytosanitary products, applicators
Biologic components needed for production	Insects, pheromones, auxiliary fauna, traps
Containers and packaging	Plastic, wood, cardboard, and netting
Preservation equipment	Refrigeration and pre-cooling
Greenhouse infrastructures	Construction components
Agricultural machinery	Tractors, machinery, and tools
Plastics	Plastic components used in greenhouses
Seeds	Research and development
Seedbed cultivators	Germination and transformation of seeds
Professional services	Consulting, biotechnology, and legal services
Environmental control systems	Ventilation, refrigeration, and heating
Fertigation systems	Tubing, driplines, sprinklers, valves
Substrates	Sand, hydroponics, fertilizers
Transportation	Activities related with transportation
Waste management	Collection and treatment

Source—Own Compilation.

**Table 2:** Conditions affecting the analyzed factors of auxiliary companies.

Competitiveness Factors
Quality of product Quality of services linked with sales Market knowledge Differentiation of product in local market High productivity of the production team Technological competitiveness Implementation of innovations Design and creativity Internationalization Training
**Competing Needs**
Increase of production capacity Increase of productivity Research and development (R&D) Adaptation of products to market demands Better logistics and distribution management Better qualified sales staff Better management information system Greater availability of financial resources Improvement of infrastructures Search of new markets
**Improvement of Competitiveness**
Purchase of machinery Introduction of innovations (products, processes) Payment of debts Distribution of profits between members Expansion of the enterprises Information systems Market research Training of employees Reshaping of the organization chart of the company Business cooperation

Source—Own compilation.

## Appendix A

**Table A1:** Results and Statistical Deviation: Infraestructures.

	Infrastructures of Greenhouses	Statistical Deviation	Result
	Competitiveness Factors		
COMPETITIVENESS FACTORS	Quality of product	2.66	7.33
	Quality of services linked with sales	2.59	5.50
	Market knowledge	2.53	5.00
	High productivity of the production team	2.51	6.50
	Differentiation of product in local market	3.27	7.33
	Technological competitiveness	1.64	5.50
	Implementation of innovations	3.13	5.17
	Training	3.20	5.67
	Internationalization	3.89	3.50
	Design and creativity	2.17	3.50
COMPETING NEEDS	Adaptation of products to market demands	1.97	6.33
	Technological development (R&D)	2.66	6.67
	Increase of productivity	3.52	7.00
	Better logistics and distribution management	0.82	5.33
	Greater availability of financial resources	3.31	5.17
	Better qualified sales staff	1.97	5.33
	Search of new markets	3.39	6.50
	Increase of production capacity	2.86	5.83
	Better management information system	4.07	4.83
	Improvement of infrastructures	1.37	2.33
IMPROVEMENT OF LOCAL COMPETITIVENESS	Training of employees	0.55	8.50
	Market research	2.14	7.17
	Introduction of innovations (products, processes)	2.26	6.50
	Business cooperation	0.98	6.83
	Expansion of the enterprise	2.94	4.33
	Purchase of machinery	3.39	4.50
	Payment of debts	1.87	3.50
	Information systems	3.61	5.67
	Reshaping of the organization chart of the company	3.25	5.83
	Distribution of profits between members	1.47	2.17

**Table A2:** Results and Statistical Deviation: Machinery

	Machinery	Statistical Deviation	Result
	Competitiveness Factors		
COMPETITIVENESS FACTORS	Quality of product	2.07	5.67
	Quality of services linked with sales	1.55	6.00
	Market knowledge	2.86	7.83
	High productivity of the production team	2.80	4.67
	Differentiation of product in local market	3.01	5.33
	Technological competitiveness	2.40	7.17
	Implementation of innovations	2.42	7.33
	Training	2.74	2.50
	Internationalization	2.07	3.33
	Design and creativity	2.56	4.17
COMPETING NEEDS	Adaptation of products to market demands	3.56	4.50
	Technological development (R&D)	2.40	8.17
	Increase of productivity	2.73	7.33
	Better logistics and distribution management	1.76	6.50
	Greater availability of financial resources	3.27	6.33
	Better qualified sales staff	2.66	4.50
	Search of new markets	3.20	4.67
	Increase of production capacity	3.01	6.33
	Better management information system	1.17	3.83
	Improvement of infrastructures	0.98	2.83
IMPROVEMENT OF LOCAL COMPETITIVENESS	Training of employees	0.52	8.33
	Market research	2.53	5.00
	Introduction of innovations (products, processes)	1.21	9.33
	Business cooperation	1.97	6.33
	Expansion of the enterprise	2.14	6.83
	Purchase of machinery	1.75	6.67
	Payment of debts	0.82	6.33
	Information systems	0.75	2.17
	Reshaping of the organization chart of the Company	1.03	2.67
	Distribution of profits between members	0.52	1.33

**Table A3:** Results and Statistical Deviation: Machinery: Plastic.

	Plastics	Statistical Deviation	Result
	Competitiveness Factors		
COMPETITIVENESS FACTORS	Quality of product	3.03	7.80
	Quality of services linked with sales	2.17	5.80
	Market knowledge	2.51	4.40
	High productivity of the production team	2.24	8.00
	Differentiation of product in local market	0.84	5.80
	Technological competitiveness	2.74	6.00
	Implementation of innovations	3.63	6.80
	Training	2.30	4.40
	Internationalization	2.07	2.60
	Design and creativity	3.29	3.40
COMPETING NEEDS	Adaptation of products to market demands	2.07	7.60
	Technological development (R&D)	1.41	9.00
	Increase of productivity	1.14	8.60
	Better logistics and distribution management	2.39	6.20
	Greater availability of financial resources	1.82	3.60
	Better qualified sales staff	1.14	5.60
	Search of new markets	4.12	4.00
	Increase of production capacity	1.82	4.60
	Better management information system	1.95	3.60
	Improvement of infrastructures	1.10	2.20
IMPROVEMENT OF LOCAL COMPETITIVENESS	Training of employees	2.49	7.20
	Market research	2.49	6.20
	Introduction of innovations (products, processes)	3.46	8.00
	Business cooperation	2.28	5.20
	Expansion of the enterprise	1.82	6.60
	Purchase of machinery	3.35	6.20
	Payment of debts	2.59	4.80
	Information systems	2.41	3.60
	Reshaping of the organization chart of the Company	2.88	5.40
	Distribution of profits between members	1.30	1.80

**Table A4:** Results and Statistical Deviation: Seedbed Cultivator

	Seedbed Cultivators	Statistical Deviation	Result
	Competitiveness Factors		
COMPETITIVENESS FACTORS	Quality of product	0.00	10.00
	Quality of services linked with sales	0.00	9.00
	Market knowledge	0.55	7.60
	High productivity of the production team	1.14	5.60
	Differentiation of product in local market	0.84	7.20
	Technological competitiveness	1.64	2.80
	Implementation of innovations	1.14	3.60
	Training	0.89	1.60
	Internationalization	2.30	3.60
	Design and creativity	1.00	4.00
COMPETING NEEDS	Adaptation of products to market demands	1.34	9.40
	Technological development (R&D)	0.45	8.80
	Increase of productivity	3.85	5.40
	Better logistics and distribution management	1.64	3.80
	Greater availability of financial resources	0.84	6.20
	Better qualified sales staff	0.84	7.20
	Search of new markets	2.30	5.60
	Increase of production capacity	1.64	3.80
	Better management information system	0.00	1.00
	Improvement of infrastructures	1.10	3.80
IMPROVEMENT OF LOCAL COMPETITIVENESS	Training of employees	1.95	7.40
	Market research	2.24	9.00
	Introduction of innovations (products, processes)	0.71	3.00
	Business cooperation	1.52	6.60
	Expansion of the enterprise	1.64	7.20
	Purchase of machinery	2.49	5.80
	Payment of debts	0.89	5.60
	Information systems	2.92	7.00
	Reshaping of the organization chart of the company	0.55	1.40
	Distribution of profits between members	0.84	1.80
